# External Validation of Pretreatment Pathological Tumor Extent in Patients with Neoadjuvant Chemoradiotherapy Plus Surgery for Esophageal Cancer

**DOI:** 10.1245/s10434-019-08024-0

**Published:** 2019-11-05

**Authors:** Sebastian Brinkmann, Bo J. Noordman, Arnulf H. Hölscher, Katharina Biermann, David van Klaveren, Elfriede Bollschweiler, Katharina Pütz, J. Jan B. van Lanschot, Uta Drebber

**Affiliations:** 1 Department of General, Visceral and Cancer Surgery, University of Cologne, Cologne, Germany; 2 Department of Surgery, Erasmus MC – University Medical Centre, Rotterdam, The Netherlands; 3 Centre for Esophageal and Gastric Surgery, AGAPLESION Markus Krankenhaus, Frankfurt, Germany; 4 Department of Pathology, Erasmus MC – University Medical Centre, Rotterdam, The Netherlands; 5 Department of Public Health, Erasmus MC – University Medical Centre, Rotterdam, The Netherlands; 6 Institute of Pathology, University of Cologne, Cologne, Germany

## Abstract

**Background:**

This study was conducted to validate a pretreatment (i.e. prior to neoadjuvant chemoradiotherapy) pathological staging system in the resection specimen after neoadjuvant chemoradiotherapy for esophageal cancer. The study investigated the prognostic value of pretreatment pathological T and N categories (prepT and prepN categories) in both an independent and a combined patient cohort.

**Methods:**

Patients with esophageal cancer treated with neoadjuvant chemotherapy and esophagectomy between 2012 and 2015 were included. PrepT and prepN categories were estimated based on the extent of tumor regression and regressional changes of lymph nodes in the resection specimen. The difference in Akaike’s information criterion (ΔAIC) was used to assess prognostic performance. PrepN and ypN categories were combined to determine the effect of nodal sterilization on prognosis. A multivariable Cox regression model was used to identify combined prepN and ypN categories as independent prognostic factors.

**Results:**

The prognostic strength of the prepT category was better than the cT and ypT categories (ΔAIC 7.7 vs. 3.0 and 2.9, respectively), and the prognostic strength of the prepN category was better than the cN category and similar to the ypN category (ΔAIC 29.2 vs. − 1.0 and 27.9, respectively). PrepN + patients who became ypN0 had significantly worse survival than prepN0 patients (2-year overall survival 69% vs. 86% in 137 patients; *p *= 0.044). Similar results were found in a combined cohort of 317 patients (2-year overall survival 62% vs. 85%; *p *= 0.002). Combined prepN/ypN stage was independently associated with overall survival.

**Conclusions:**

These results independently confirm the prognostic value of prepTNM staging. PrepTNM staging is of additional prognostic value to cTNM and ypTNM. PrepN0/ypN0 patients have a better survival than prepN +/ypN0 patients.

**Electronic supplementary material:**

The online version of this article (10.1245/s10434-019-08024-0) contains supplementary material, which is available to authorized users.

Potentially curative treatment for esophageal cancer consists of neoadjuvant chemo(radio)therapy followed by surgery. After neoadjuvant therapy, the percentage of residual tumor cells and lymph node regression is of prognostic value. Several studies investigated the impact of tumor regression and classified histopathological response to neoadjuvant therapy and its correlation to prognosis.^1^^–^4 Prior to treatment, clinical staging is known to be relatively unreliable,^1^^,^^5^ particularly for the N category, and an improvement of the pretreatment stage is needed.^6^

Recently, Shapiro et al.^7^ introduced a new staging system based on the pretreatment (i.e. prior to neoadjuvant chemoradiotherapy) pathological tumor extent, which is determined by the extent of regressional changes and the presence of residual tumor cells in the resection specimen. These regressional changes were hypothesized to reflect the pretreatment tumor extent. The authors proved this so-called ‘pretreatment pathological T and N staging’ (prepT and prepN categories) to be estimated reproducibly, with high concordance between three upper gastrointestinal pathologists from different institutes (intraclass correlation coefficient of between 0.7 and 0.9). It was demonstrated that the prognostic strength of the prepT category is comparable with the pretreatment clinical T category (cT category, according to the Union for International Cancer Control [UICC] TNM Cancer Staging, 7th edition^7^), while the prognostic strength of the prepN category is even better than the pretreatment clinical N category (cN category), and better predicts overall survival than the post-treatment pathological N category (ypN category) alone.

The primary aim of the present study was to externally validate the pretreatment pathological staging system of T and N stage based on the extent of regressional changes and the presence of residual tumor cells in the resection specimen. In addition, we aimed to study the prognostic value of this new staging system in the post-treatment setting by combining the pretreatment prepN category and the post-treatment ypN category, to distinguish between patients who were lymph node-negative before neoadjuvant chemoradiotherapy and patients who became lymph node negative thanks to neoadjuvant chemoradiotherapy.

## Methods

### Patient Selection

Between November 2012 and April 2015, patients treated with curative intent for esophageal or junctional cancer, who underwent neoadjuvant chemoradiotherapy according to the CROSS regimen, and who had an en bloc transthoracic esophagectomy with intrathoracic reconstruction (Ivor–Lewis procedure) at the Department of General, Visceral and Cancer Surgery, University of Cologne (Chairman at that time: Professor Dr. A.H. Hölscher) were included in this study. Patients with non-epithelial tumors and other types of esophageal resection and reconstruction were excluded. According to the recently presented study protocol,^1^ patients who did not receive at least 80% of the planned dose of neoadjuvant chemoradiotherapy and who received a different neoadjuvant chemoradiotherapy regimen, or patients with an intraoperatively unresectable tumor, were excluded. Patients who had < 80% of the planned dose of neoadjuvant chemoradiotherapy were also excluded because these patients have limited response due to dose reduction, and not due to tumor biology. However, the group of patients who had < 80% of neoadjuvant chemoradiotherapy was < 2% of all patients and therefore likely does not influence the results. Patients did not participate in the CROSS trial. The protocol of the present study was approved by the Ethics Committee of the University Hospital of Cologne (reference number 16-266).

### Clinical Staging and Surgery

Clinical staging consisted of a standardized preoperative work-up, including endoscopy with histological biopsy, endoscopic ultrasonography (EUS), and thoracic and abdominal computed tomography (CT). Clinical T and N categories were determined by EUS and CT scanning according to the UICC TNM Cancer Staging, 7th edition.^7^ Patients were classified as cN + or cN −.  For EUS, the criteria for lymph node involvement were a short axis diameter of ≥ 6 mm, a specified hypoechoic pattern or spherical contour and distinct border. On CT, lymph nodes were considered involved if the short axis measurement was ≥ 1 cm, located in the expected distribution, demonstrated altered density or enhancement, and a loss of the fatty hilum could be observed. All patients received neoadjuvant chemoradiotherapy 41.4 Gy and carboplatin/paclitaxel according to the CROSS regimen.^8^^,^^9^ The standard surgical procedure of esophagectomy comprised laparoscopic or open abdominal lymphadenectomy and gastric tube formation, right-sided anterolateral thoracotomy with an en bloc esophageal resection, and two-field lymph node dissection. Reconstruction was performed with a high intrathoracic stapled esophagogastrostomy. The complete surgical approach has been described in detail elsewhere.^10^^,^^11^

### Post-treatment Pathological Staging

The resection specimens (primary tumor and all resected lymph nodes) were removed en bloc and analyzed in accordance with a standardized protocol.^12^ Histopathological tumor characteristics were scored using the UICC TNM Cancer Staging, 7th edition.^7^ The adapted Mandard scoring system was used to determine the tumor regression grade (TRG).^13^

### Pretreatment Pathological Staging

All resection specimen slides of all patients were examined by two pathologists from the Department of General Pathology and Pathological Anatomy at the University of Cologne. In case of disagreement between the two pathologists, consensus was achieved by consensus discussion. After having proven interobserver agreement of pathological estimations of pretreatment primary tumor extent and lymph node involvement with high reproducibility of prepT and prepN staging in the resection specimen at the Department of Pathology Rotterdam, a meeting of pathologists from both centers was organized to improve validation of the pretreatment pathological staging system. In accordance with Shapiro et al.,^5^ the original tumor region, before neoadjuvant chemoradiotherapy, was estimated based on the extent of regressional changes (e.g. fibrosis, mucinous lakes, keratin pearls, and/or foreign body giant cell reactions) and the presence of residual tumor in the resection specimen.^8^^,^^12^^,^^14^ The ‘pretreatment pathological T category’ (prepT category), reflecting the estimated original invasion depth of the primary tumor, was based on the extent of regressional changes in the esophageal wall and peri-esophageal stroma. In addition, interpretation of the ‘pretreatment pathological N category’ (prepN category), reflecting the estimated number of originally involved lymph nodes, was dependent on the presence of regressional changes in lymph nodes. Lymph nodes that showed complete regression (based on pathological examination) without the presence of residual tumor were considered to have been sterilized by neoadjuvant chemoradiotherapy. PrepT and prepN staging were scored using the UICC TNM Cancer Staging, 7th edition.^7^

### Data Collection and Follow-Up

Data were collected from a prospectively maintained database. All patients were regularly evaluated during follow-up, with 3-month intervals within the first year, 6-month intervals within the second year, and an annual aftercare from the third year onwards. Survival was determined by using hospital records. Overall survival was calculated from the day of surgery until the date of death from any cause.

### Statistical Analysis

Data were described using medians and interquartile ranges (IQRs) in the case of continuous variables, or frequencies with percentages in the case of categorical variables.

Prognosis and prognostic strength were based on overall survival data. The difference between Akaike’s information criterion of the model and the null model (ΔAkaike’s information criterion) was calculated to measure the prognostic strength of a model.^15^ A higher ΔAkaike’s information criterion value indicates better prognostic ability, adjusted for the statistical complexity of the model fit. It is calculated by the likelihood ratio (LR) Chi square statistic of the corresponding Cox proportional hazards model minus two times the degrees of freedom. PrepN and ypN categories were analyzed as ordinal variables and continuous variables. For models with continuous variables, restricted cubic splines with three knots (corresponding with two degrees of freedom) were used. Kaplan–Meier plots were used to depict survival, and the log-rank test was applied to assess survival differences.

By combining the prepN and ypN categories, patients were divided into three groups: (1) patients without nodal involvement before and after neoadjuvant chemoradiotherapy (prepN0/ypN0); (2) patients with nodal involvement before neoadjuvant chemoradiotherapy and no detectable lymph node involvement after neoadjuvant chemoradiotherapy (prepN +/ypN0); and (3) patients with nodal involvement before neoadjuvant chemoradiotherapy remaining node-positive even after neoadjuvant chemoradiotherapy (prepN +/ypN +). To determine the independent association between the combined prepN and ypN categories and overall survival, a multivariable Cox regression model was used. Clinicopathological characteristics, which are known as prognostic factors (i.e. age, sex, histology, TRG, and ypT stage), were included in the multivariable model. All reported *p* values are two-sided and *p* values < 0.05 were considered statistically significant. Statistical analysis was performed using SPSS 21 for Windows (IBM Corporation, Armonk, NY, USA).

## Results

### Clinical and Histopathological Characteristics

Overall, 137 patients were included in this study. Clinicopathological characteristics of patients are displayed in Table 1. Median age at the time of surgery was 62 years; 110 patients were male (80%), 97 patients had an esophageal or junctional adenocarcinoma (71%), and 40 patients had an esophageal squamous cell carcinoma (29%). The majority of patients was clinically staged as cT3 (90%) and cN + (88%). In addition, 97 patients were staged prepT3 (71%) and 82 patients were staged prepN + (60%). The median number of resected lymph nodes was 28, with an IQR of 22–35.

**Table 1:** Clinical and histopathological characteristics of 137 patients with esophageal or junctional cancer treated with neoadjuvant chemoradiotherapy according to the CROSS trial, plus extended surgical resection

	*n*	%^a^
Age, years		
Median (p25–p75)	62 (57–68)	
Sex		
Female	27	20
Male	110	80
Tumor type		
Squamous cell carcinoma	40	29
Adenocarcinoma	97	71
cT category		
cT1	2	1
cT2	10	7
cT3	123	90
cT4	2	1
cN category		
cN0	17	12
cN-positive	120	88
prepT category		
prepT1	14	10
prepT2	26	19
prepT3	97	71
prepN category		
prepN0	55	40
prepN1	34	25
prepN2	35	26
prepN3	13	9
Number of nodes resected		
Median (p25–p75)	28 (22–35)	
ypT category		
ypT0	40	29
ypT1	24	18
ypT2	22	16
ypT3	51	37
ypN category		
ypN0	81	59
ypN1	21	15
ypN2	27	20
ypN3	8	6
Tumor regression grade		
TRG1	40	29
TRG2	40	29
TRG3	32	23
TRG4	24	18
TRG5		–
Missing	1	

*TRG* tumor regression grade

^a^Data are expressed as median (interquartile range) or number (%). Percentages may not add up to 100 because of rounding. The Mandard scoring system was used to determine the TRG^15^

### Comparison of PrepT Category with cT and ypT Categories

**Fig. 1:**
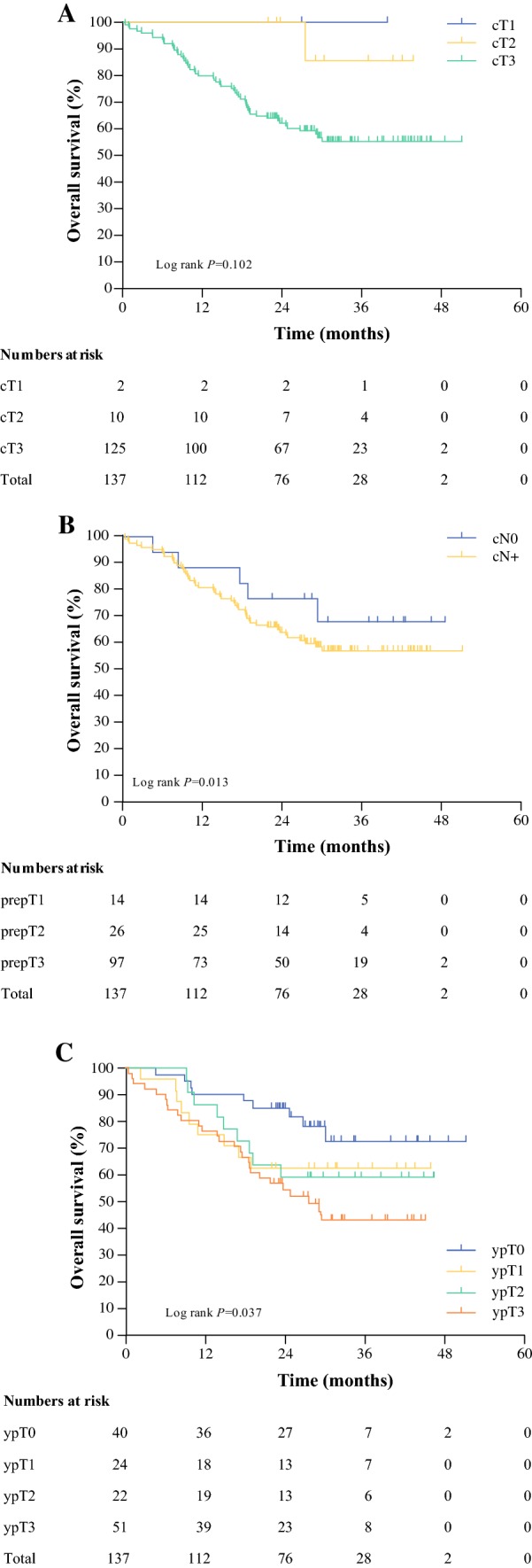
Overall survival according to **a** clinical T category, **b** pretreatment pathological T category, and **c** post-treatment pathological T category in 137 patients

Non-concordant prepT categories (compared with the cT category) were found in 41 of 137 patients (30%). With regard to the cT category, 37 patients were found to be overstaged. Four patients had a less advanced prepT category compared with the cT category (Table 2a). The prognostic strength of the prepT category was higher compared with the cT and ypT categories (ΔAkaike’s information criterion 7.7 vs. 3.0 and 2.9, respectively) [Table 3]. Overall survival curves according to the cT, prepT, and ypT categories are shown in Fig. 1.

**Table 2:** Comparison of clinical (a) T and (b) N categories (cT and cN categories) with pretreatment pathological T and N categories (prepT and prepN categories) in 137 patients

		PrepT category			Total
		1	2	3	
(a)					
cT category	1	2	0	0	2
	2	3	3	4	10
	3	9	23	91	123
	4	0	0	2	2
Total		14	26	97	137

		PrepN category				Total
		0	1	2	3	
(b)						
cN category	Negative	9	5	3	0	17
	Positive	46	29	32	13	120
Total		55	34	35	13	137

**Table 3:** Prognostic stratification based on pretreatment clinical T category, pretreatment pathological T category, and post-treatment pathological T category, and pretreatment clinical N category, pre treatment pathological N category, and post-treatment pathological N category

	Data type	LR Chi square	*df*	ΔAIC^a^	c-statistic (SE)
T staging					
cT category	Ordinal	7.0	2	3.0	0.55 (0.02)
prepT category	Ordinal	11.7	2	7.7	0.60 (0.03)
ypT category	Ordinal	8.9	3	2.9	0.61 (0.04)
N staging					
cN category	Ordinal	1.0	1	− 1.0	0.52 (0.02)
prepN category	Ordinal	35.2	3	29.2	0.71 (0.04)
ypN category	Ordinal	33.5	3	27.5	0.69 (0.04)
prepN category	Continuous	33.9	2	29.9	0.71 (0.04)
ypN category	Continuous	31.1	2	27.1	0.68 (0.04)
prepN + ypN categories	Continuous	35.4	4	27.4	0.71 (0.04)

^a^This measure represents the prognostic strength of a model and is calculated by the LR Chi square statistic of the corresponding Cox proportional hazards model minus two times the *df*. A higher ΔAIC value indicates better prognostic ability, adjusted for the statistical complexity of the model fit^17^

*ΔAIC* difference between Akaike information criterion of the model and the null model, *LR* likelihood ratio, *df* degrees of freedom, *c*-*statistic* concordance statistic, *SE* standard error

### Comparison of PrepN Category with cN and ypN Categories

**Fig. 2:**
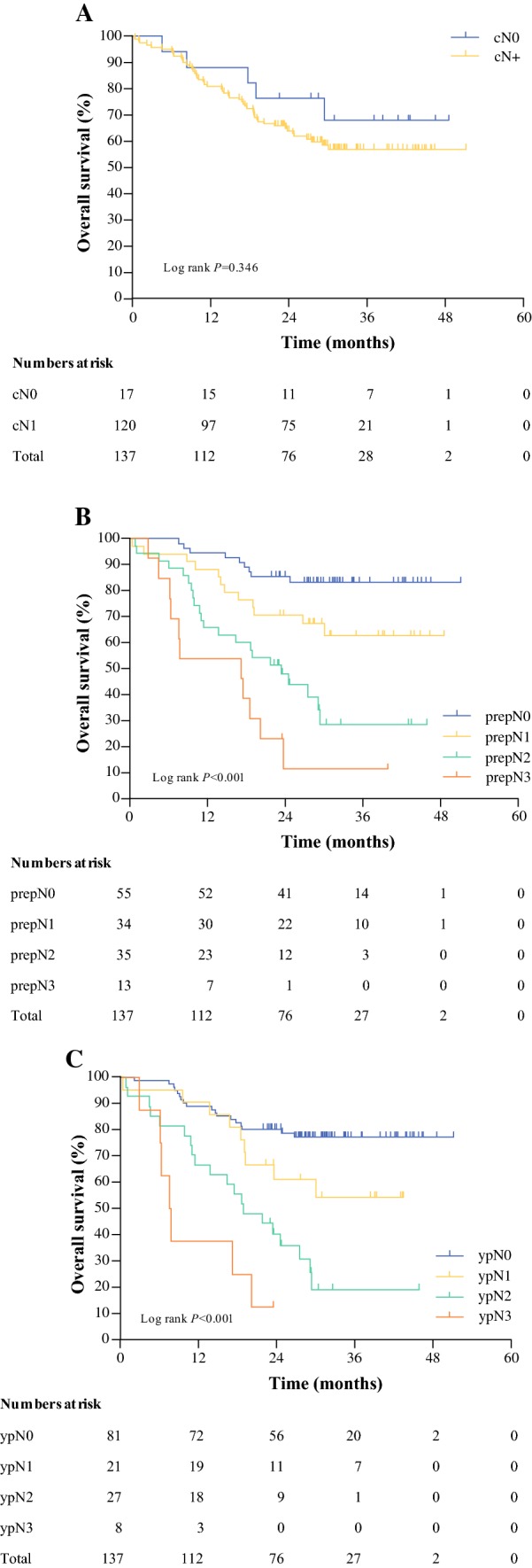
Overall survival according to **a** pretreatment clinical N category, **b** pretreatment pathological N category, and **c** post-treatment pathological N category in 137 patients

Non-concordant prepN categories (compared with the cN category) were found in 54 of 137 patients (39%). Overall, 8 patients were clinically staged cN0, but showed pathological signs of pretreatment nodal involvement in the resection specimen. In contrast to this, 46 patients were clinically staged cN +, but pathological signs of pretreatment nodal involvement could not be observed (Table 2b). The prognostic strength of the prepN category was better than the cN category, and similar to the ypN category (ΔAkaike’s information criterion 29.2 vs. − 1.0 and 27.9, respectively) [Table 3]. The overall survival curves according to the cN, prepN, and ypN categories are shown in Fig. 2.

### Combining PrepN and ypN Categories

**Fig. 3:**
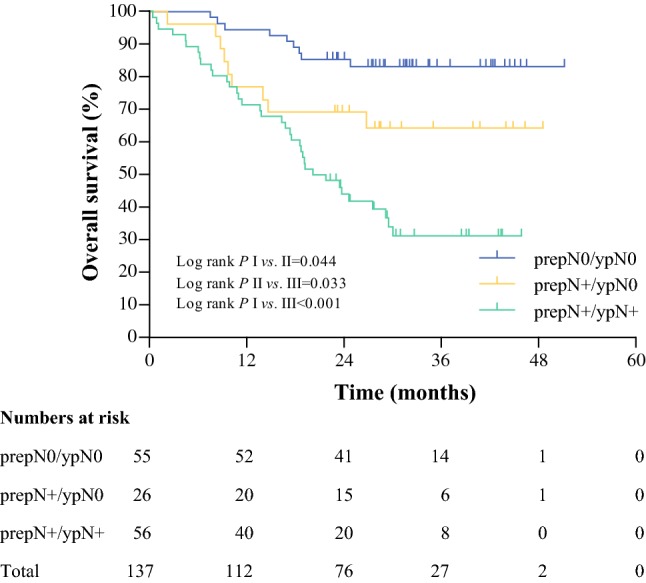
Overall survival according to the combined scoring of pretreatment pathological N category and post-treatment pathological N category in 137 patients. Groups I, II, and III represent prepN0/ypN0, prepN +/ypN0, and prepN +/ypN +, respectively

Two-year overall survival in patients without nodal involvement before and after neoadjuvant chemoradiotherapy (group 1) was 86%, compared with 69% in patients with nodal involvement before neoadjuvant chemoradiotherapy who became node-negative after neoadjuvant chemoradiotherapy (group II) (*p* = 0.004) [Fig. 3]. Patients who remained node-positive even after neoadjuvant chemoradiotherapy (group III) had the worst survival (2-year OS = 44%) compared with group I (*p* < 0.001). Differences between groups were statistically significant (*p *< 0.001). Patients who had a nodal involvement pretreatment, but became node-negative after neoadjuvant chemoradiotherapy, had a statistically significantly better 2-year overall survival compared with patients who remained node-positive after neoadjuvant chemoradiotherapy (69% vs. 44%; *p *= 0.003).

### Multivariable Analysis in a Combined Patient Cohort

**Fig. 4:**
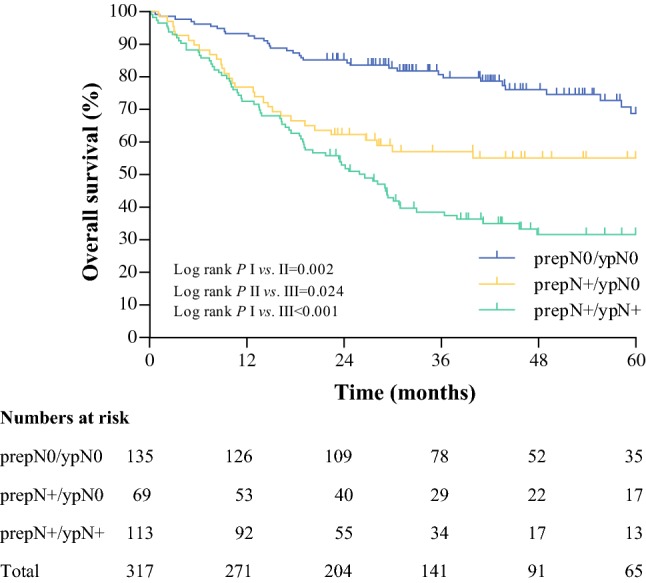
Overall survival according to the combined scoring of pretreatment pathological N category and post-treatment pathological N category in 317 patients from Cologne and Rotterdam. Groups I, II, and III represent prepN0/ypN0, prepN +/ypN0, and prepN +/ypN +, respectively

Finally, data of all patients from the present study (*n *=137), and from the previously reported Rotterdam Study (*n *=180),^7^ were combined and served as the basis of a multivariable model to prove combined prepN and ypN categories as an independent factor of prognosis. The overall survival curves are shown in Fig. 4. Entering the combined prepN and ypN categories in the multivariable model could identify this additional staging parameter as an independent prognostic factor for overall survival (prepN +/ypN +; hazard ratio [HR] 2.84, 95% confidence interval [CI] 1.82–4.44; *p * < 0.01) [Table 4].

**Table 4:** Multivariable Cox regression analysis of prognostic factors related to survival in 317 patients with esophageal cancer

	HR	95% CI	*p* value
Age	1.03	1.01–1.05	**0.02**
Sex			
Male	1 (ref)	–	–
Female	0.67	0.42–1.05	0.08
Histology			
Squamous cell carcinoma	1 (ref)	–	–
Adenocarcinoma	0.91	0.59–1.41	0.68
ypT category			
ypT0	1 (ref)	–	–
ypT1	0.84 (ref)	0.23–3.01	0.79
ypT2	0.71	0.19–2.65	0.61
ypT3/4	1.00	0.28–3.59	1.00
Mandard			
1	1 (ref)	–	–
2	2.08	0.58–7.38	0.26
3	2.36	0.63–8.88	0.20
4	2.41	0.63–9.17	0.20
Combined prepN and ypN			
prepN0/ypN0	1 (ref)	–	–
prepN +/ypN0	2.17	1.31–3.58	**< 0.01**
prepN +/ypN+	2.84	1.82–4.44	**< 0.01**

Bolded *p* values are statistically significant (i.e. *p *< 0.05)

Analyses of the prognostic value of overall prepTNM and ypTNM stage grouping showed comparable prognostic strength of prepTNM (classified according to the cTNM classification system) and ypTNM stage grouping (ΔAIC 41.2 and 40.0, respectively; supplemental analyses).

## Discussion

The present study was conducted to validate the recently presented pretreatment pathological staging system, based on the extent of regressional changes and on the presence of residual tumor cells in the resection specimen.^5^ In an independent cohort of patients treated for esophageal cancer with neoadjuvant chemoradiotherapy followed by surgery at another high-volume center, it was shown that the pathological estimations of the prepT and prepN categories in the resection specimen have a high prognostic power and can therefore be implemented in the pathological assessment.

The present study further aimed to prove the prognostic value of the pretreatment pathological staging system in the post-treatment setting. We confirmed that the prepT and prepN categories have a better prognostic strength than the clinical T and N categories. This proves the association of this new staging parameter with postoperative overall survival.

Shapiro et al. found that the prognostic strength of the prepT category is similar to the clinical T category, but worse compared with the ypT category (ΔAkaike’s information criterion 1.3 vs. 2.0 and 8.9, respectively), and the prognostic strength of the prepN category is better than the cN category, but similar to the ypN category (ΔAkaike’s information criterion 17.9 vs. 6.2 and 17.2, respectively). In the present study, the prognostic strength of the prepT category was even better than the ypT category. Furthermore, we found the prepN category to have a better prognostic strength than the cN category.

However, comparing the results of both studies, it has to be mentioned that in the present study, clinical staging of lymph node involvement only differentiated patients in cN + and cN0. Of 17 clinically node-negative patients, 8 patients (47%) showed pathological signs of pretreatment nodal involvement, explaining the low ΔAkaike’s information criterion of the cN category (− 1.0) compared with the prepN and ypN categories. In the study by Shapiro et al.,^7^ 37% of patients were clinically staged falsely negative with regard to lymph node involvement. The results of both studies demonstrate the poor N-staging accuracy of CT, EUS, and positron emission tomography (PET)/CT scanning, emphasizing that the clinical estimation of nodal involvement in the preoperative setting is unreliable. This is in line with previous studies reporting similar poor radiological cN staging accuracy,^6^^,^^16^^–^18 with a sensitivity and specificity of CT, EUS, and PET/CT of 39.7% and 77.3%, 42.6% and 75%, and 35.3% and 90.9%, respectively.^17^

Our study confirms that patients who did not have any pretreatment nodal involvement (prepN0) have a better prognosis than patients who had no residual disease in the resected lymph nodes (ypN0), but who did have pretreatment nodal involvement (prepN +).^5^ This is in contrast with Donohoe et al.,^3^ who found that clinically node-positive patients who had complete nodal response had no difference in survival compared with initially clinically node-negative patients. This is probably due to the low accuracy of clinical N staging. However, in the present study, multivariable analysis proved combined prepN and ypN categories as an independent factor of prognosis. Patients staged prepN + who became ypN0 after neoadjuvant chemoradiotherapy had a significantly worse survival compared with prepN0 patients, with a 2-year overall survival of 62% vs. 85% (*p *=0.002) in the combined group of 317 patients. These findings are in concert with the results of a previously presented study by Nieman et al.,^19^ who found a negative prognostic impact of initial nodal involvement even after complete response after neoadjuvant chemoradiotherapy. By staging lymph nodes negative, with no viable cancer cells but the evidence of tumor necrosis in the resection specimen after neoadjuvant chemoradiotherapy, the current TNM staging system is deficient. The previously published data from Shapiro et al.,^5^ along with the present results, confirm this thesis as the prognostic value of pretreatment pathological staging of lymph nodes is superior to the conventional post-treatment pathological assessment, and patients staged prepN + have a significant worse survival compared with patients staged prepN0.

The question of clinical relevance of pretreatment pathological staging focuses on the impact of adjuvant therapy in the case of prepN + patients. Recently, Hsu and colleagues studied the benefit of adjuvant treatment in patients with persistent nodal involvement or with an increasing T category (non-responders) after neoadjuvant chemoradiotherapy, and found an improvement in disease-free survival in non-responders treated after neoadjuvant chemoradiotherapy, with a significantly reduced rate of systemic recurrence.^20^ For the purpose of sterilizing subclinical lymph node metastases or micrometastases by adjuvant therapy in prepN + patients, but not in prepN0 patients, this staging system can achieve clinical significance.^21^ Further studies, based on the proposed pretreatment pathological staging, should examine the benefit of adjuvant treatment between different groups of patients according to the prepN categories.

Limitations of the present study include the inclusion of patients with both squamous cell carcinomas and adenocarcinomas. These subtypes are biologically different; however, both respond to neoadjuvant chemoradiotherapy and no statistically significant differential effects were found in the CROSS trial. Moreover, there was no interaction with histological subtype (*p* for interaction = 0.63), suggesting that the effect of the combined prepN and ypN categories is not modified by histology. In the recently introduced 8th edition of the TNM staging system, adenocarcinoma and squamous cell carcinoma are classified differently. Moreover, this revised edition accounts for a new cTNM classification, based on actual clinical stage (rather than re-iterating the pTNM system based on patients who had surgery alone), and a new ypTNM system based on patients receiving neoadjuvant therapy. In the current paper the (obsolete) 7th edition of the TNM staging system was used as this paper is an external validation of a previous study. Therefore, identical methods were applied.^5^ Furthermore, the sample size is relatively limited but was sufficient to validate the initial study and to show that the combined prepN/ypN stage is an independent prognostic factor.

## Conclusions

These results independently confirm the previously described prognostic value of the pretreatment pathological staging system. Pretreatment pathological staging is of additional prognostic value to both cTNM and ypTNM and should be considered as a new staging parameter.

## Electronic supplementary material

Below is the link to the electronic supplementary material.

Supplementary material 1 (DOCX 67 kb)
