# Supplementary material for: External Validation of Pretreatment Pathological Tumor Extent in Patients with Neoadjuvant Chemoradiotherapy Plus Surgery for Esophageal Cancer
Source: Ann Surg Oncol. 2019 Nov 5;27(4):1250–8. doi: 10.1245/s10434-019-08024-0 (PMC7060166; doi:10.1245/s10434-019-08024-0)
Supplement: Supplementary file 1 — Supplementary material 1 (DOCX 67 kb) [file 10434_2019_8024_MOESM1_ESM.docx]

**Supplemental Figure 1.** Overall survival according to prepTNM-(figure 1a, log rank p <0.001) and ypTNM-stage groups (figure 1b, log rank <0.001), according to the Union for International Cancer Control (UICC) TNM Cancer Staging, 8^th^ edition. Prognostic strength of both models was comparable (ΔAIC 41.2 and 40.0, respectively).


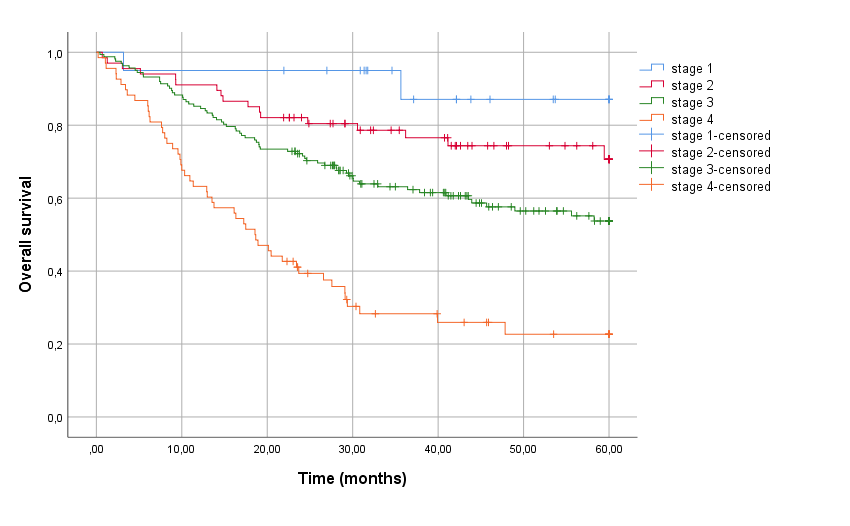


A

B
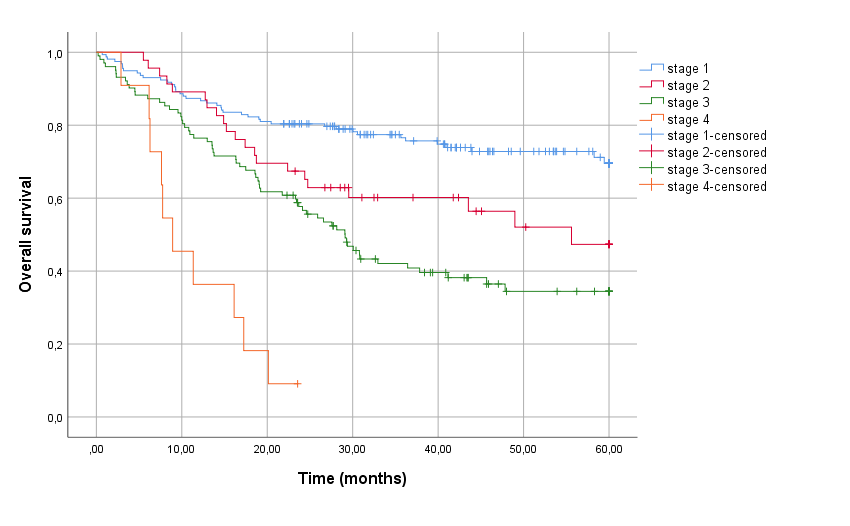


ΔAIC: difference between Akaike information criterion of the model and the null-model.17 This measure represents the prognostic strength of a model and is calculated by the likelihood ratio χ2 statistic (LR χ2) of the corresponding Cox proportional hazards model minus two times the degrees of freedom (df). A higher ΔAIC value indicates better prognostic ability, adjusted for the statistical complexity of the model fit.
